# Supplementary material for: Acceptance and compliance with micronutrient powder (MNP) among children aged 6–23 months in northern Nigeria
Source: PLOS Glob Public Health. 2022 Oct 17;2(10):e0000961. doi: 10.1371/journal.pgph.0000961 (PMC10022258; doi:10.1371/journal.pgph.0000961)
Supplement: S1 Dataset — (ZIP) [file pgph.0000961.s008.zip › S8_Dataset/Spot Checks.zip!/Spot Checks/0972015_A_GA_PM_2_SC.pdf]

SPOT CHECK RECORDING FORM

State: __ADAMAWA______________ LGA: _GANYE________________ Community: ___SUGU__________________
NAME OF CAREGIVER(FEMALE)MAGDALENE MOSES…………………………………………….

Sex of index child: _M_____________ Age of index child (in months): __7________________

Spot Check Information

1. **Date of MNP distribution to household** (circle option below based on LGA name above)**:**

   - **Kebbi:** BirninKebbi&Jega LGA: **June 16**
   - **Kebbi:** Danko-wasagu: **June 17**
   - **Adamawa:**Song, Ganye: **June 18**
   - **Adamawa:**Guyuk **June 19**

2. **Date of Spot Check Observation: __9-07-2015_________________**

3. **Number of sachets remaining**(Ask the caregiver to show you and count the specific number of sachets remaining)**:**

   41

4. **Number of sachets that should remainusing one MNP sachet per day** (Analyst only)**:**

   40

5. **Number of sachets remaining / number of sachets should be remaining**(Analyst only)**:**

____41_____ / ____40_____ = _102.5______ %

Probes

Question 1. You have **XX** MNP sachets remaining. Explain how easy or difficult it was to use the MNP product on a daily basis for your child 6 – 23 months? (Circle one answer)

Very Difficult **Difficult** **Easy** **Very Easy**

Question 2. Describe any specific challenges that you faced that made the MNP difficult to use on a daily basis?NONE AT ALL

Question 3. Describe specific aspects of the MNP product that you or your child enjoyed that made it easier to use on a daily basis? THE CHILD EATS MORE MEAL THAN BEFORE………HE HAS ADDED MORE STRENGHT AND WEIGHT…….

Question 4. What questions do you have about the MNP based on your experience using it?

DOES THE MNP POWDER HELPS DESTROY WORMS IN A CHILD.?

Question 5. What specific recommendations do you have that we can do as a program to make the MNP easier to use on a daily basis? YOU CAN DEVELOP A JINGL ON RADIO THAT WILL KEEP REMINDING MOTHERS ON A DAILY BASIS,SECONDLY,THEY CAN INVOLVE THE RELIGIOUS LEADERS,SO THAT EVERY SUNDAY DURING WORSHIP,THEY CAN ALWAYS PASS AN ANNOUNCEMENTS REMINDING MOTHERS.
